# Gemvid, an open source, modular, automated activity recording system for rats using digital video

**DOI:** 10.1186/1740-3391-4-10

**Published:** 2006-08-25

**Authors:** Jean-Etienne Poirrier, Laurent Poirrier, Pierre Leprince, Pierre Maquet

**Affiliations:** 1 Cyclotron Research Center, University of Liege, Allee du 6 Aout, 8 (B30), 4000 Liege, Belgium; 2 Centre for Cellular and Molecular Neurobiology, University of Liege, Avenue de l'Hôpital, 1 (B36), 4000 Liege, Belgium; 3 Applied Sciences Faculty, University of Liege, Chemin des Chevreuils, 1 (B52), 4000 Liege, Belgium

## Abstract

**Background:**

Measurement of locomotor activity is a valuable tool for analysing factors influencing behaviour and for investigating brain function. Several methods have been described in the literature for measuring the amount of animal movement but most are flawed or expensive. Here, we describe an open source, modular, low-cost, user-friendly, highly sensitive, non-invasive system that records all the movements of a rat in its cage.

**Methods:**

Our activity monitoring system quantifies overall free movements of rodents without any markers, using a commercially available CCTV and a newly designed motion detection software developed on a GNU/Linux-operating computer. The operating principle is that the amount of overall movement of an object can be expressed by the difference in total area occupied by the object in two consecutive picture frames. The application is based on software modules that allow the system to be used in a high-throughput workflow. Documentation, example files, source code and binary files can be freely downloaded from the project website at .

**Results:**

In a series of experiments with objects of pre-defined oscillation frequencies and movements, we documented the sensitivity, reproducibility and stability of our system. We also compared data obtained with our system and data obtained with an Actiwatch device. Finally, to validate the system, results obtained from the automated observation of 6 rats during 7 days in a regular light cycle are presented and are accompanied by a stability test. The validity of this system is further demonstrated through the observation of 2 rats in constant dark conditions that displayed the expected free running of their circadian rhythm.

**Conclusion:**

The present study describes a system that relies on video frame differences to automatically quantify overall free movements of a rodent without any markers. It allows the monitoring of rats in their own environment for an extended period of time. By using a low-cost, open source hardware/software solution, laboratories can greatly simplify their data acquisition and analysis pipelines and improve their workload.

## Background

Measurement of locomotor activity is a valuable tool for analysing factors influencing behaviour and for investigating brain function. As a result, assessment of locomotor activity has been used in many fields such as neurotoxicology, psychopharmacology, biological rhythm research, etc.

In the past, the most widely used automated devices for measuring locomotor activity have been stabilimeters [1], microwaves [2], photocell-based systems [3] and running wheels [4]. The latter technology has gradually come to dominate the area, probably because of its reasonable cost and adaptability to varying environmental configurations. But these methods in general either present flaws or are of very high cost. For example, counting interruptions of infrared light beams in a cage using photocell sensor units mainly reflects locomotion instead of overall movements and has poor temporal resolution. The reconstruction of multi-dimensional movements from markers tracked by motion analysis systems is not suitable for measuring overall movements (because markers are placed on the entire body and these markers prevent free animal behaviour) and is costly. Running-wheel systems fail to record activity when the animal is not on the wheel and may induce changes in circadian period [5,6]. Finally, a simple, visual observation of behaviour and manual counting of movements is subjective and prone to inter-examiner differences.

Newer technologies have become available that provide the opportunity to detect motor activity, primarily locomotion, in a different and potentially more accurate way. Some of these are contrast-sensitive or frame-difference video tracking systems [7-9]. In addition to the use of the frame-difference technique, the system we present here takes advantage of two other technological evolutions: increasing computer power, allowing the use of inexpensive technologies that were previously costly (e.g. IR-camera that can record even in the dark) and open source software, widely and openly available to anyone for use and modification.

We designed our system based on four ideal, basic requirements [10]:

• Data collection should not influence the rhythmic physiological variables.

• Acquired data should permit flexible and powerful analysis.

• Data should be collected regularly during each oscillatory period (increasing frequency spectrum) and for many successive cycles (increasing frequency resolution).

• Data collection should be automated.

## Methods

Our system consists of a closed-circuit television (CCTV) camera that non-invasively records all movements of a rat in its own cage. It then uses custom-designed motion detection software running on a GNU/Linux-based personal computer. The operating principle is that the amount of overall two-dimensional movement of an animal can be expressed by the difference in total area occupied by the object in two consecutive picture frames (two-dimensional object-difference method [7]), during light and dark periods.

**Figure 1:**
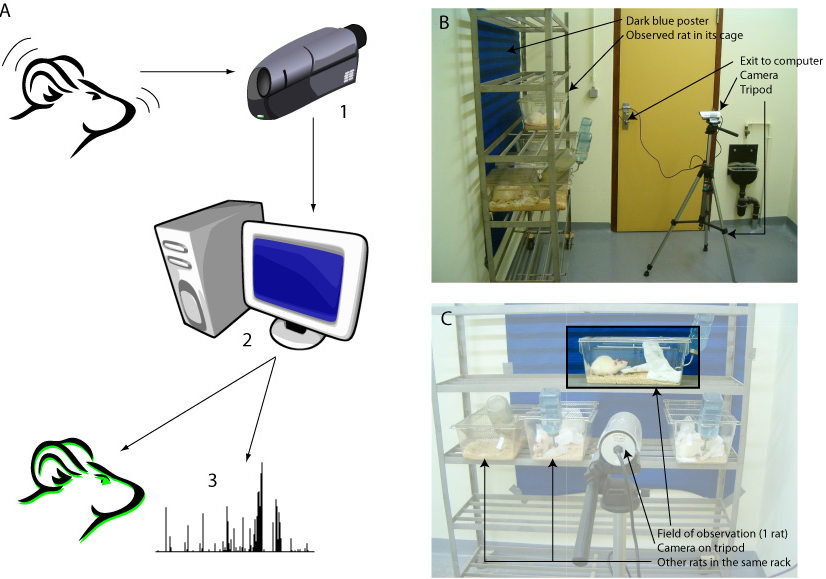
**The three components of the video image analysis system**. A: schematic representation; B and C: picture of the setup in our laboratory.

The video image analysing system consists of 3 components (Figure 1): a visible/infrared CCTV camera, a computer equipped with a common TV tuner and modular software pieces.

For the first component, we used a small, low-cost CCTV camera (LYD-806C CCD, Lianyida, China), capable of working at 0 lux and mounted on a standard tripod. This construction allowed the adjustment of the height of the camera and the adjustment of the angle and distance between the camera and the animal cage. The camera was set in such a way that the longest side of the rat cage was perpendicular to the camera view. In this configuration, we could measure more animal movements. In the light phase, light was provided by three 133 cm, 36 W neon tubes placed on the ceiling. They produced a perceived intensity of 260lux in the cage (LX-6610 luxmeter, Elix). During the dark phase, 30 infrared LEDs inside the camera were automatically switched on.

Conventional equipment was used for the last hardware component: an Intel Pentium II personal computer running RedHat Linux v.7. This computer uses a video card with a TV tuner/frame grabber (Rage 128 Pro, ATI, USA).

Images from the camera were transmitted to the computer and digitised by the frame-grabber. Our system digitises 25 frames per second (25 Hz). Frame resolution is 360 by 240 pixels (86400 pixels in total). Pixel size is 0.133 cm at 1 m.

Any other camera/frame grabber system can be used, provided it is recognized by the Video4Linux library . This allows the use of commercial webcams, regular (IR-unable) camera and CCTV.

The computer programs were designed with modularity in mind. Each module can be used separately. Moreover, results from each module are openly described and can be used by any other custom process, software or analyser. A clear advantage is that, by launching the same module in different processes, our system can easily be extended to simultaneously monitor several animals. Software are written in C and licensed under the GNU General Public Licence. Documentation, example files, source code and binary files can be freely downloaded from the project website at .

Our first software module acquires frames and compares each frame with the previous one. The number of pixels that change between two frames is associated with the time during which the frame was taken (when activity occurred: in hour, minute, second and millisecond). Our system processes 25 frame comparisons per second (25 Hz, as fast as frames arrive).

The software first shows the observation field in real time and overlays a layer that highlights the changed pixels in green. Thus, a first use of this software allows visualization of behavioural changes that occur in real time.

A signal can be sent to the operator or back to the rat environment if the amount of movement is below a user-defined threshold (number of pixels) for a user-defined duration. This signal can be, for instance, visual (on a computer screen) and/or auditory (through computer loudspeakers).

Numerical results from the first module are sent to the standard output: every 25th fraction of a second, a string containing the time and the number of pixels changed, compared to the previous frame, is sent to the command line. The number of modified pixels is an indirect measure of rat overall activity. This output allows the quantification of changes that occur in real time.

Via a pipe or a redirection, data output from the first module can be stored in a text file. The data output can be easily modified to store values in other formats. The length of a continuous data acquisition period is limited only by the memory size of the computer hard disk (a day of acquisition is contained in a 80 Mb text file; 6 Mb when compressed with gzip).

Data can later be processed by a second software module. The current module draws actograms, indicating the relative intensity of the overall movement of the rat (y-axis) at a given time (x-axis). Other analyses can be performed by any mathematical or statistical software package.

Two other parameters can be set: a minimum and/or maximum count value. The minimum count value sets the minimum number of changed pixels that are scored as movement. In this experiment, we set the lower limit filter value at 100 pixels, corresponding approximately to a square of 2 cm^2 ^in our experimental setup.

In experiment 1, we investigated the sensitivity and reproducibility of our device and software to detect the exact frequency of movement of a small object with regular movement. We placed a metronome (Taktell Piccolino, Wittner, Germany) at a distance of 1 meter from the camera and we recorded several series of oscillations at different frequencies (40, 52, 100, 152 and 200 oscillations per minute). Each recording lasted 60 seconds. We applied a short-time Fourier transform (in Matlab R2006a, Mathworks, USA) to recorded signals in order to extract observed frequencies. We also performed a Pearson's product-moment correlation test between theoretical and observed frequencies (R 2.3.1, R Foundation for Statistical Computing, Austria [11]).

In experiment 2, we left the metronome (at 52 oscillations per minute) in front of the system during 10 hours. Since the metronome signal is stable in time, we tested the detection stability of our system. We also applied a short-time Fourier transform to compare spectrograms obtained at the beginning and at the end of the test. This procedure allowed us to detect any potential drift of signal detection in time, leading to false increase or decrease in movement.

In experiment 3, we were interested in the sensitivity of our system and in the comparison of our data with another well-established activity-monitoring device. On a custom-designed mobile going at two different speeds (23.81 cm · s^-1 ^and 45.45 cm · s^-1^), we placed an Actiwatch Plus (Cambridge Neurotechnology Ltd, United Kingdom) and a white square paper of variable surface. This paper was placed perpendicularly in front of the camera. We recorded a series of movements of the mobile at different speeds and with different surface areas (80, 128, 160, 192, 224 and 256 cm^2^). This area range was chosen because it encompassed the area occupied by the projection of a rat seen laterally on a vertical surface (approximately 150 cm^2^). We reported data obtained from our system and from the Actiwatch on the same chart. A Pearson's product-moment correlation test was also applied to recorded signals with different moving areas.

Experiments 4 to 6 were performed on 8 male Sprague-Dawley rats weighing 200–250 g at the time of observation. Upon their arrival, the rats were housed in group cages and had food and water ad libitum. Seven days prior to observation, rats were individually housed in smaller cages (18 cm high × 29 cm wide × 20 cm deep), food and water still ad libitum. The room was maintained at a temperature of 22–24°C and a relative humidity of 30–40%. All procedures were approved by the Ethics Committee of the University of Liège.

In order to maintain a certain level of animal welfare during data acquisition, we left the rat in its own cage, at the same place in the cage rack, with a little bit of nesting material. Nothing was placed inside the rat environment. A dark blue poster was left hanging on the wall behind the rat cage. This provided a sufficient contrast with white rats when viewed in visible light.

On the day before the first day of observation, the camera was placed approximately at 2 m from the back wall of the cage. The observation frame contained the whole cage. Apart from the animal, all other objects remained inert in the observation field.

In experiment 4, we investigated the sensitivity of our device with real animals and the types of movement that it effectively detected. Data acquisition was started simultaneously with the time-stamped video-cassette recording of the rat (AG-VP320, Panasonic, Japan). Later on, the video-cassette was played back and we compared the amount of pixels that had changed with the observed behaviour.

In experiment 5 (6 rats), a 12 h light-dark cycle was imposed with the lights automatically turned on at 06:00 h and off at 18:00 h (LD 12:12). The rats were left undisturbed for the next 7 days with food and water ad libitum, except on day 3, when litter was changed and food and water were provided when necessary. Data were automatically and continuously collected for the whole experimental period, except when we changed the litter. Subsequently, data analysis was performed off-line. Our data analysis included the data presentation with the second software module (creation of actogram) and a test of stability over time. This test was also performed in order to verify that there was no drift in the detection system. For that purpose, we calculated the mean number of pixels that changed each hour. The means between the days and conditions (light or dark) were compared using an ANOVA in R.

Experiment 6 (2 rats) was carried out with two rats in the same conditions as in experiment 5 except that the rats were kept in constant darkness (DD, 0 lux) since the first day of observation. Data presentation was performed with the second software module.

## Results

**Figure 2:**
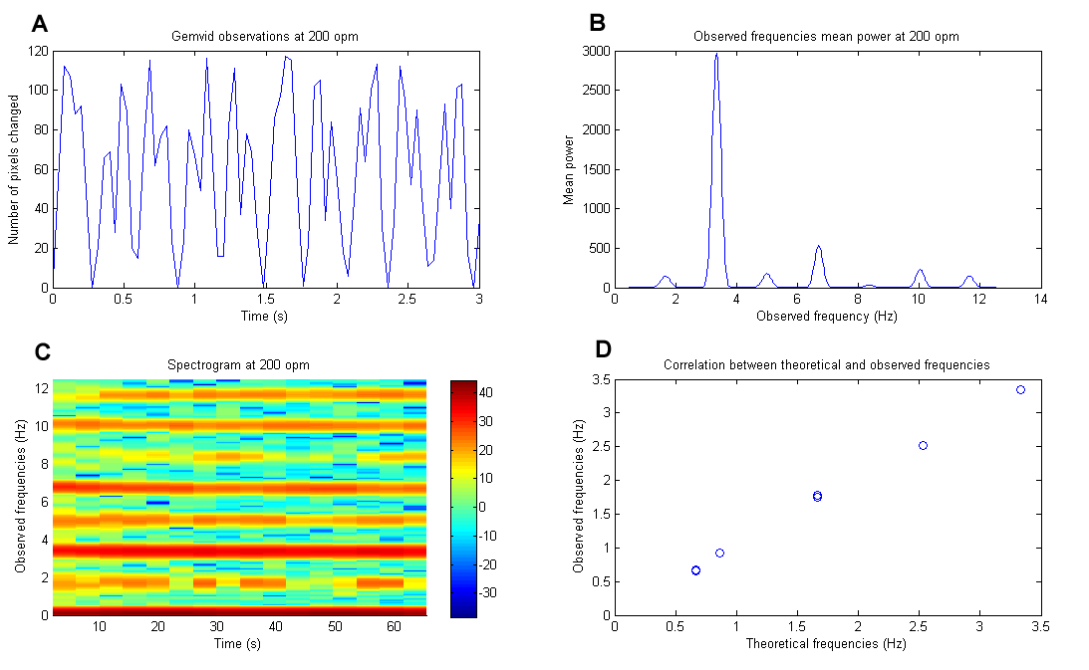
**Observation and spectral analysis of a metronome at 200 oscillations per minute**. A: observations from the first software module. B: mean power of observed frequencies; 3.35 Hz is the frequency that has the maximum mean power (200 opm = 3.333 Hz). C: spectrogram of observed frequencies over time (intensity scale on the right) (opm = oscillations per minute). D: correlation plot between theoretical and observed frequencies of a metronome (n = 24)

In the first experiment, the system was able to collect enough samples to find the right oscillation frequencies of the metronome. Figure 2 (A, B, C) shows the result of the spectral analysis for one frequency (200 oscillations per minute, or 3.333 Hz). In Figure 2 (D), we plot the observed frequencies versus theoretical frequencies (set up on the metronome from 0.66 to 3.3 Hz). Pearson's correlation coefficient is 0.9992245 (p < 10^-16^), indicating that the observed frequencies are not statistically different from the theoretical ones.

**Figure 3:**
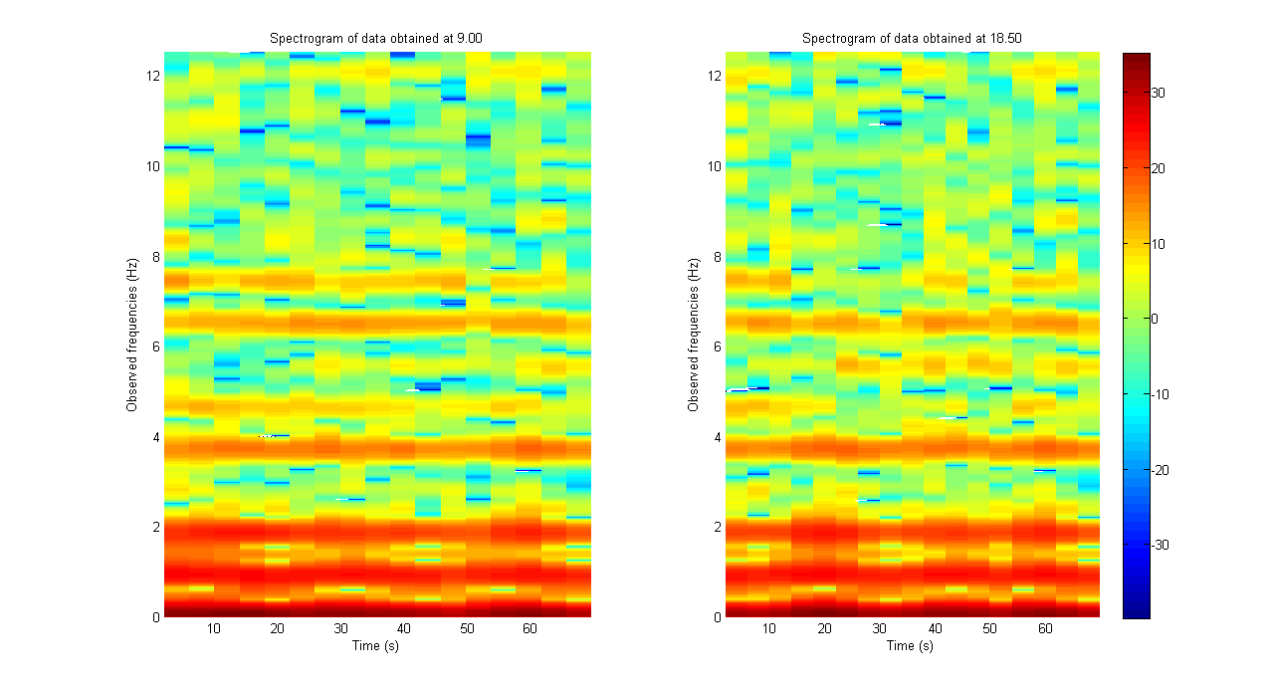
**Spectral analysis of two periods of data acquisition with a metronome set up at 52 oscillations per minutes = 0.8667 Hz, in a continuous experiment**. Left: spectrogram from data acquired at 9:00 hr. Right: spectrogram from data acquired at 18:50 hr.

In Figure 3, we show two spectrograms derived from observed oscillations of the metronome (at 52 oscillations per minute, or 8.86667 Hz) at the beginning and end of a 10-hour continuous experiment. The comparison of the two spectrograms shows that there is no drift of detected signal after 10 hours: the main frequency coefficient of variation during the first quartile is 0.32701% and this coefficient of variation during the last quartile is 0.57637%.

**Figure 4:**
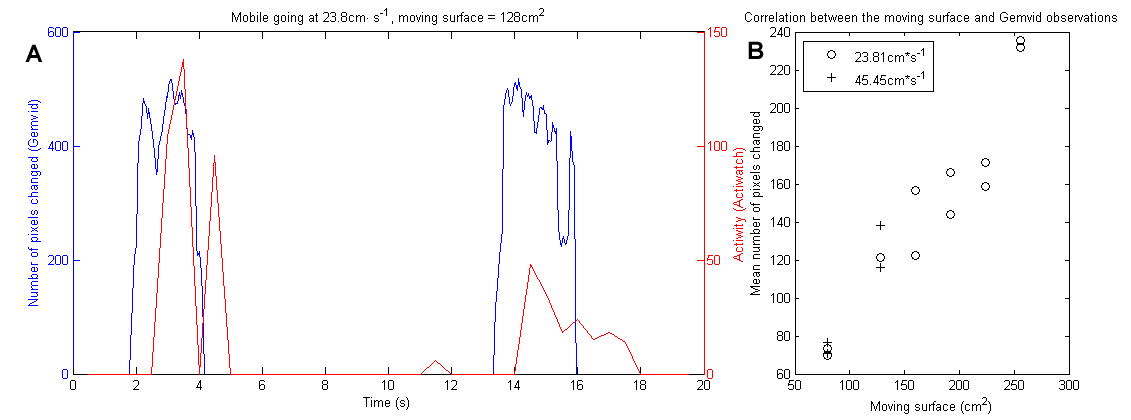
**Comparison of data from Gemvid and from Actiwatch**. A: observed data from each device is presented in blue for Gemvid and red for Actiwatch. Mobile is going at 23.8 cm · s^-1 ^and the mobile surface is 128 cm · s^-1^. B: correlation plot between the size of the moving surface and the mean number of pixels that changed for each value of area (n = 3 for each value of area).

The comparison of data collected with our system and data from the Actiwatch show that they are quite similar. However, our system detected activity before the Actiwatch (example of two movements of our mobile in Figure 4A). Three factors made this comparison difficult. First, the Actiwatch resolution is low as its shortest period is 2 s while our system's shortest period is 0.04 s. Second, observed movements in this type of setup are in the lower range of sensitivity for the Actiwatch. Finally, the Actiwatch can only detect variation in speed of movement ("activity") while our system also detects variations in the amount of movement (number of changed pixels, see next paragraph). In the same third experiment, we compared the number of pixels that changed using different moving areas. In Figure 4B, we plot the mean number of pixels changed versus different areas. Pearson's correlation coefficient is 0.9511966 (p < 10^-7^), indicating that the size of a body that moves (at the same speed) is related to the number of pixels that change.

**Figure 5:**
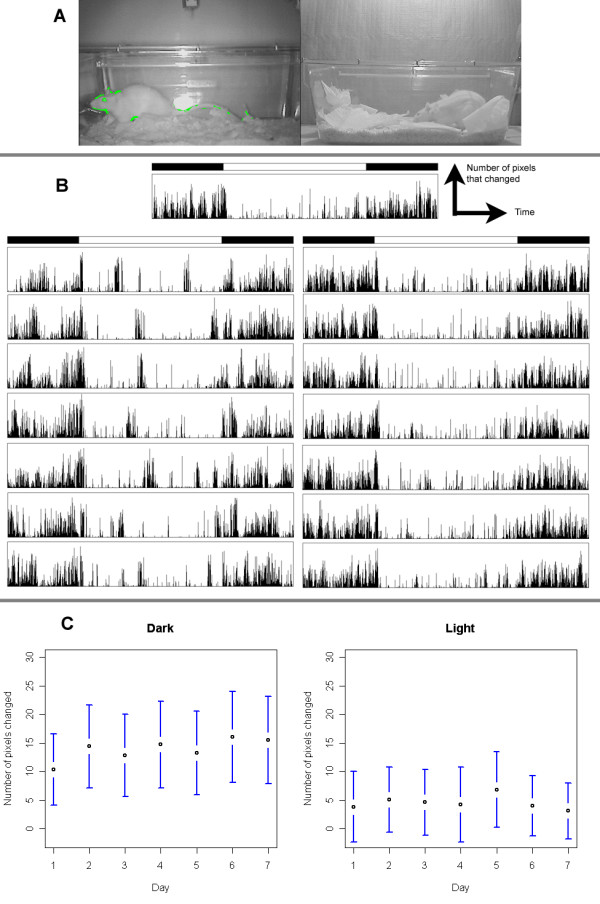
**Data from software module 1**. A: screenshot showing the actual view of software module 1. Green pixels highlight zones that changed since the last frame. Movements highlighted here were only observed around the head and the tail. Left: the rat is active; right: the rat is sleeping. B: Examples of actograms. Top: one day actogram for rat #1 (LD 12:12 with lights on at 6:00). Below: seven days actogram for rat #3 (left) and #6 (right). C: mean number of pixels changed each day for one rat (left: in dark conditions ; right: in light conditions).

In the fourth experiment, the amount of pixels that changed during the data acquisition was compared with the rat behaviour. We observed that the system was very sensitive, even to very small movements of the head or the tail (Figure 5A, left). When rats were sleeping, no movement was detected (Figure 5A, right).

The recordings of 7 days of activity of 6 different rats in the second experiment were similar to those presented in Figure 5B, top. Actograms are plots of the number of pixels changed (y-axis) versus time during the day (x-axis). Figure 5B, bottom left, shows an interesting activity pattern: for unknown reasons, rat 3 recurrently increased its activity around noon (other rats did not show the same activity pattern; nothing in the environment could explain this specific behaviour).

In Figure 5C, we plot the mean number of changed pixels each day for one rat. The ANOVA test indicates that there is a significant difference between the two conditions, as expected (p < 0.001) but that there is no significant day effect nor difference in the interaction between day and condition (p < 0.05). All this indicates that there is no drift in the detection system over time.

**Figure 6:**
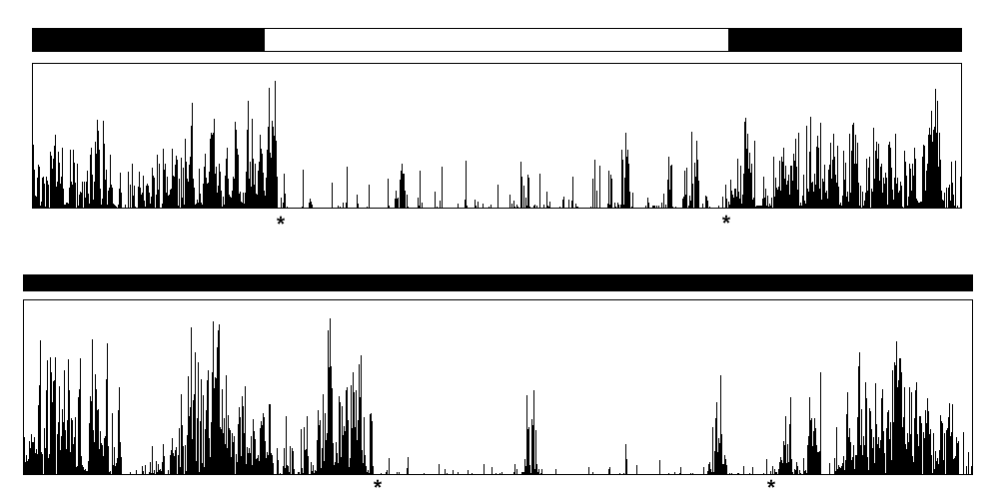
**Activity of a rat in L:D and D:D conditions**. Top: 3rd day in LD conditions ; bottom: 5th day under free-running, DD conditions ; a * denotes change in activity.

Finally, Figure 6 compares the activity of the same rat between a regular day in LD conditions and the fifth day in DD conditions. In LD conditions, the rat drastically reduced its activity at 6:24 (24 minutes after lights were switched on) but became very active just after the light were switched off (at 18:00). Four days after being put in DD conditions, it drastically reduced its activity at 10:02 (4 hours after the initial drop in activity) but became very active only at 19:44 (nearly two hours after lights were switched off in the previous configuration).

## Conclusion

The present study describes a system that relies on frame difference video technology. The main operating principle is that the amount of overall movement of an object can be expressed by the total area of the object which changes from frame to frame. We demonstrate that the system we designed is sensitive enough and stable in its acquisition process.

To test the validity of this system, we first tested it with a metronome, giving a regular and stable movement. We show that the system is sensitive enough to detect the small movements of the metronome at 1 m of distance and that the observed data contains the oscillation frequency. We demonstrate that there is no drift in the detection process since observed data contain the same oscillation frequencies for at least 10 hours. Moreover, we show that, while recording a real animal, the mean number of pixels that changed each day was also not significantly different.

We have compared our system with an Actiwatch device and, despite some limiting factors inherent to the Actiwatch, both systems recorded activity at approximately the same time. Moreover, we also showed that, with our system, there was a strong correlation between the mean number of pixels changed and the size of the areas moving at the same speed.

To test this system with animals, rats were observed in LD and DD conditions. The results of experiment 4 show that there is usually more activity during the dark periods than the light periods, as expected for a nocturnal animal. Our system was also able to detect changes in the length of the endogenous circadian period. Indeed, *Rattus norvegicus *has a period of more than 24 h that becomes apparent in free-running conditions (constant darkness here).

We overcame the limitation of video recording during darkness by using a camera that, by automatically switching to infrared in complete darkness, allowed us to continuously monitor activity during the light and dark phases.

There are, however, three disadvantageous features of this technique:

• The recording of movements parallel to the camera viewpoint axis (vertical movements when the camera is above the cage) cannot be recorded by a single camera. Certain movements within the region of the object cannot be detected, such as movements in the direction of the camera (it is for this reason that we placed the longest side of the cage in front of the camera).

• Part of the bedding that are actively displaced by the rodent during foraging sometimes lead to miscalculation of the overall movement. This issue might be solved by including a segmentation step before the frame substraction.

• Finally, one should note that our system can record activity from only one animal at a time. This limitation can be overcome by including a segmentation step or, for a small number of animals, by launching as many processes as different animals/cameras in the experiment.

In summary, this method has many interesting advantages:

• all instruments are commercially available at comparatively low cost (a Pentium II computer can be found for less that US$ 90.00 and our CCTV costs US$ 70.00);

• the analysis procedure is performed automatically, and no skill is necessary;

• placed outside the animal environment, the data collection system does not influence the rhythmic physiological variables;

• the overall movements of animals can be estimated with high sensitivity;

• our system can easily be extended to the simultaneous monitoring of several animals;

• acquired data allows flexible and powerful analysis as well as a full integration in a bigger data collection framework (use of open formats).

These advantageous features make the Gemvid system a powerful device for overall movement assessment in rodents.

## Competing interests

The author(s) declare that they have no competing interests.

## Authors' contributions

LP wrote the first module software and built the mobile device for the third experiment. JEP wrote the second module software, carried out the experiments, data analysis and prepared the successive versions of the manuscripts. PL and PM supervised the experiments. The study was conceived and planned by JEP. All authors approved the final version of the manuscript
